# Seroconversion and dynamics of IgG anti-SARS-CoV-2 antibodies during the pandemic: A two-month observation cohort study on the population of Sleman in Indonesia

**DOI:** 10.1371/journal.pone.0316360

**Published:** 2025-01-02

**Authors:** Jajah Fachiroh, Septi Kurnia Lestari, Dewi Kartikawati Paramita, Bagas Suryo Bintoro, Fatwa Sari Tetra Dewi, Lutfan Lazuardi, Cici Permata Rusadi, Erti Nur Sagenah, Eggi Arguni

**Affiliations:** 1 Department of Histology and Cell Biology, Faculty of Medicine, Public Health and Nursing, Universitas Gadjah Mada, Yogyakarta, Indonesia; 2 Sleman Health and Demographic Surveillance System, Faculty of Medicine, Public Health and Nursing, Universitas Gadjah Mada, Yogyakarta, Indonesia; 3 Department of Health Behavior, Environment and Social Medicine, Faculty of Medicine, Public Health and Nursing, Universitas Gadjah Mada, Yogyakarta, Indonesia; 4 Center of Health Behavior and Promotion, Faculty of Medicine, Public Health and Nursing, Universitas Gadjah Mada, Yogyakarta, Indonesia; 5 Department of Health Policy and Management, Faculty of Medicine, Public Health and Nursing, Universitas Gadjah Mada, Yogyakarta, Indonesia; 6 Department of Child Health, Faculty of Medicine, Public Health and Nursing, Universitas Gadjah Mada, Yogyakarta, Indonesia; 7 Center for Tropical Medicine, Faculty of Medicine, Public Health and Nursing, Universitas Gadjah Mada, Yogyakarta, Indonesia; Murdoch Children’s Research Institute, AUSTRALIA

## Abstract

**Background:**

This study describes the seroconversion and serodynamics of IgG antibodies against the RBD of SARS-CoV-2 in the general population of Sleman District, Yogyakarta Special Province. We aim to identify possible factors that correlate with the seroconversion and serodynamics of IgG antibodies against the RBD of SARS-CoV-2.

**Methods:**

We performed a longitudinal study of the population at Health and Demographic Surveillance System (HDSS) Sleman, Yogyakarta, Indonesia. Study subjects were recruited between April and December 2021 using convenience sampling and were followed up 2 times, i.e. 4–5 and 8–9 weeks. The inclusion criteria for subjects were age ≥ 18 years, absence of flu-like symptoms, and negative COVID-19 by using GeNose C19^®^ screening. A community-based survey on demographics, comorbidities and smoking habits were documented at baseline, while a history of vaccination, COVID-19-related symptoms, mobility, and preventive measures, weight and height as well as a venous blood draw, were collected at each visit. The anti-RBD-SARS-CoV-2 IgG antibody concentration from blood plasma was measured using chemiluminescent microplate immunoassay (CMIA). Descriptive analysis was performed based on IgG seropositivity by using chi-squared test or Fisher’s exact test, as appropriate. Logistic regression was subsequently performed to identify factors that were correlated with IgG seropositivity. Further, a grouping of subjects based on IgG seropositivity was done to analyze factors that might correlate with seroconversion and serodynamics of anti-RBD-SARS-CoV-2 IgG antibody. A P value ≤ 0.05 was considered to indicate a significant difference.

**Results:**

Three hundred eighty-five (385) participants were analyzed. At baseline, 307 out of 385 (79.7%) subjects were seropositive for the IgG antibody against the RBD of SARS-CoV-2. Descriptive analysis showed that sex, marital status, smoking habits, obesity, vaccination status, and preventive measures were different between the IgG anti-RBD-SARS-CoV-2 seropositive and negative individuals (p≤ 0.05). Further analysis showed that, vaccination was the factor most strongly correlated with seropositivity [OR = 20.58; 95% CI 10.82, 39.15]. Based on the correlation, we separated subjects into 4 groups. Group 1 (seronegative-unvaccinated individuals; 50 subjects); Group 2 (seronegative-vaccinated individuals; 27 subjects); Group 3 (seropositive-unvaccinated individuals; 25 subjects); and Group 4 (seropositive-vaccinated individuals; 282 subjects). During monitoring, 27/49 (55.10%), 5/25 (20%), 9/22 (40.91%), and 27/257 (10.51%) of subjects in Group 1, 2, 3, and 4 respectively, received 1 or 2 doses of COVID19 vaccine. When comparing seroconversion at baseline and monitoring 2, positive IgG seroconversion was observed in Group 1 (from 0/51 (0%) to 23/49 (46.94%)) and Group 2 (from 0/27 (0%) to 10/25 (40%)), but negative seroconversion was observed in Group 4 (from 282/0 (100%) to 248/257 (96.50%)); while, all subjects in Group 3 remained seropositive at the end of monitoring. This evidence suggested for hybrid immunity, on which infection and vaccine simultaneously contributes to anti-RBD-SARS-CoV-2 IgG seroconversion.

**Conclusions:**

A high seroprevalence of the IgG antibody against RBD-SARS-CoV-2 in the Sleman population was found to correlate with COVID-19 vaccination and as infection occurred, thus enhancing hybrid immunity. We also identified nonresponder and rapid antibody decaying individuals, that call for targeted vaccinations in addition to annual universal boosting.

## Introduction

The WHO declared the end of the COVID-19 pandemic as early as May 2023. However, in December 2023, an increase in COVID-19 incidence was observed globally [1]. Vaccination for immunocompromised patients has been advocated, especially for probable endemic cases. Calls for expanding nonclinical health protection have been made despite the significantly lower number of documented COVID-19 cases and deaths than during the pandemic era [1].

Seroprevalence can indicate the presence of an immune response against certain pathogens and may aid in public health policy. In general, the seroprevalence of SARS-CoV-2 in the general population was underreported compared to that among individuals who presented with COVID-19 symptoms [2]. Prior to the introduction of vaccine, a number of serological surveys were carried out during the early pandemic and indicated characteristics such as age, sex, immunological state, and the test used may have been associated with the severity of the disease [3–6]. Supporting these data, two cross-sectional studies in Indonesia [7, 8] conducted before the introduction of vaccination (2020–2021) showed that the seroprevalence was greater in dense populations than in less dense populations, in slums than in non-slums, and among those of reproductive age (30–50 years old). Interestingly, a smaller study (n = 425) in the Bantul District, Yogyakarta, Indonesia, showed that the seroprevalence did not significantly differ between individuals in terms of their preventive measures and mobility [9].

A prospective study in Manaus, Brazil, involving 1,638 seronegative participants who had no COVID-19 diagnosis at recruitment, showed that they had seroconversion after 57 days (interquartile range (IQR): 54–61 days) with 48.1% asymptomatic. The risk factors for seroconversion were the incidence of COVID-19 in the family, not using a mask when in contact with someone with COVID-19, easing physical distancing, and symptoms similar to flu or COVID-19 [10].

There is a consensus that primary SARS-CoV-2 infection provides some form of protective immunity, as shown by several studies among individuals before vaccination which also reported that protective effects of natural infection can last for five to eight months [6, 11, 12]. A meta-analysis [13] reported that subjects with severe COVID-19 had greater protection against reinfection after 12 months than those with mild symptoms (74.6% vs. 24.7%). Following vaccination, antibody levels dropped after several months, resulting in a decrease in protection from infection [14–17], while still protecting against COVID-19 hospitalization. This combination of natural infection-induced immunity and vaccine-induced immunity conferred the protection is called hybrid immunity. The effectiveness of hybrid immunity against hospital admission or severe disease was 97.4% (95% CI: 91.4–99.2) at 12 months after primary series vaccination, and 95.3% (81.9–98.9) at 6 months after the first booster vaccination, after the most recent infection, or vaccination. The effectiveness of hybrid immunity against reinfection following primary series vaccination decreased to 41.8% (95% CI: 31.5–52.8) at 12 months, while the effectiveness of hybrid immunity following the first booster vaccination decreased to 46.5% (36.0%–57.3%) at 6 months [13].

**Fig 1:**
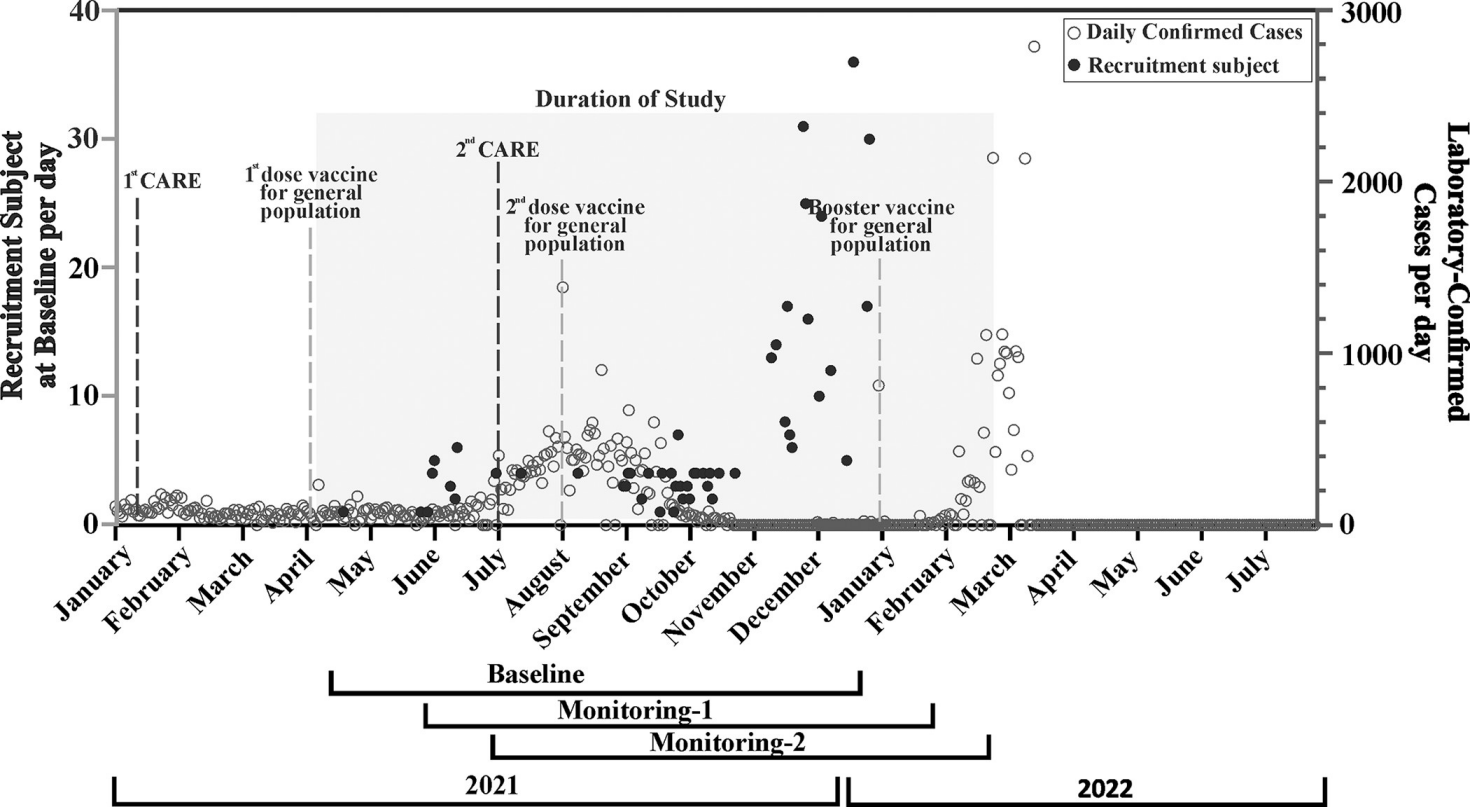
Milestones of the study. Numbers of daily subjects’ recruitment on baseline (closed dots; Y1-axis: numbers of subjects) were shown together with the number of daily newly confirmed COVID-19 cases (open dots; Y2-axis: numbers of COVID-19 cases) in Sleman District [26]. Public health measures and COVID-19 vaccination policy starting events (marked with vertical lines on the graph) are also included in the figure. The first stage of Community Activities Restrictions Enforcement (CARE) was implemented in January 2021 and the second stage of CARE was started in July 2021. Timeline of baseline, monitoring 1 (4–5 weeks post inclusion) and monitoring 2 (8–9 weeks post inclusion) is depicted at the base of the figure, ilustrating the duration of recruitment from April 2021 to Decemeber 2021, with the final monitoring completed in February 2022 (grey area).

This study aims to describe the seroconversion and serodynamics of IgG antibodies against the receptor-binding domain (RBD) of SARS-CoV-2 in the general population of Sleman, Yogyakarta Special Province, over a period of two months. This study is part of the SYNTHESIS (Surveillance System to Observe Seroconversion to SARS-CoV-2 in Human) study that was described elsewhere [18]. It was conducted during the lifting of lockdown restrictions and initiation of vaccination in 2021 (Fig 1), a time when many factors could have played a role in the seroconversion and serodynamics of IgG RBD-SARS-CoV-2 antibody levels [19]. Within this study, we observed the indication of hybrid immunity despite the short observation period.

## Materials and methods

### Study design and participants

This was a prospective cohort study on seroconversion and serodynamics among healthy individuals in the general population. It was nested within the Sleman Health and Demographic Surveillance System (HDSS), which includes the residents of Sleman District within the Yogyakarta Special Province. The utilization of a convenience sampling method was necessitated by the lockdown policy enforced during the study period. In total, 11 out of 17 subdistricts were selected. The Sleman HDSS respondents residing in these selected subdistricts who met the eligibility criteria (adults aged ≥18 years) were invited to participate in the present research’s baseline data collection. Data and sample collection were carried out at community centers within each subdistrict, strictly adhering to all COVID-19 restrictions.

All eligible individuals who accepted the invitation were subsequently screened to determine whether they met any inclusion or exclusion criteria of this study. The inclusion criteria for this study included healthy adults (≥18 years old) who agreed to participate and tested negative for SARS-CoV-2 infection by utilizing the GeNose C19^®^ (Swayasa Prakarsa, Indonesia) a noninvasive device that analyzes an individual’s breath to detect volatile organic compounds indicative of SARS-CoV-2 infection [20] prior to their interview. The GeNose C19^®^ is a breathalyzer with high potential for rapid COVID-19 screening, as it has a sensitivity of 86–94% and a specificity of 88–95%. Additionally, it does not require an invasive procedure. Furthermore, the Indonesian Ministry of Health formally acknowledged and distributed it for COVID-19 screening at the end of December 2020. Any patient’s affirmative GeNose C19^®^ result must be verified by a diagnostic test in accordance with the World Health Organization (WHO) guideline. The GeNose C19^®^ test results were provided to participants within 20 minutes. Positive results led to a referral to a public health center for further evaluation, while negative results were followed by an explanation of the research, consent collection, blood sample collection, measurement of weight and height, and a short interview (10–15 minutes).

The exclusion criteria included the presence of flu-like symptoms (such as fever, malaise, exhaustion, nausea, cough, headache, muscle aches, convulsion, skin rash, vomiting, chills, diarrhea, anosmia, and ageusia) at the time of screening, as well as the presence of immunosuppressive conditions, immune deficiency diseases, or receipt of any immunosuppressive therapy.

All study participants were subsequently invited to participate in monitoring session 1 (4 weeks after baseline) and monitoring session 2 (8 to 9 weeks after baseline collection). Both monitoring sessions followed the same procedure as the baseline data collection. Study participants were categorized as drop-out cases if they did not attend or were deemed ineligible for both of the monitoring sessions. We began recruiting participants on April 29, 2021, finished on December 16, 2021, and completed monitoring session 2 on February 21, 2022.

### Data and blood sample collection

Survey, physical measurements (height and weight), and collection of blood sample were carried out at three distinct time points: baseline, monitoring session 1, and monitoring session 2. The interviews collected data on essential demographic information (e.g., age, sex, education, employment status, marital status), smoking habits, COVID-19 vaccination status), comorbidities (diabetes, cancer, stroke, hypertension, obesity, heart disease, chronic obstructive pulmonary disease, chronic liver disease, chronic kidney disease, hemorrhagic fever, tuberculosis, human immunodeficiency virus, and autoimmune disease), history of COVID-19 symptoms, and contact with COVID-19 patients, mobility (travel history), and preventive measures (compliance with health protocols). The weight and height of the participants were measured for body mass index (BMI) calculations.

A 3-mL venous blood sample was collected from each participant using an EDTA-containing vacutainer. Blood samples were transported to the Biobank Unit at the Faculty of Medicine, Public Health, and Nursing, UGM, in Yogyakarta, Indonesia. Blood plasma was obtained through refrigerated centrifugation at 350 × g for 10 minutes. All specimens were processed and stored on the same day of receipt, and the isolated plasma was stored at -80°C until testing.

### Anti-RBD-SARS-CoV-2 IgG level

The SARS-CoV-2 IgG II Quant assay (Abbott, Ireland) was used for the quantitative determination of IgG antibodies against the receptor-binding domain (RBD) of SARS-CoV-2. The laboratory analysis was performed according to the manufacturer’s instructions. The resulting chemiluminescent reaction was measured as a relative light unit (RLU). IgG seropositivity to the RBD of SARS-CoV-2 was determined at an RLU ≥ 50 AU/mL, as written in the manual of the product.

### Ethical considerations and declarations

This study was reviewed and approved by the Medical and Health Research Ethics Committee (MHREC), of the Faculty of Medicine, Public Health, and Nursing, UGM, with reference no. KE/FK/0882/EC/2020 on August 6, 2020, and the ethical approval was extended with reference no. KE/FK/1063/EC/2021 and KE/FK/1443/EC/2022 on September 24, 2021 and November 16, 2022 respectively. Written informed consent was obtained from all respondents prior to enrollment in this study.

### Data management

The interviews were conducted by a research nurse using a digital questionnaire app installed on the study tablet PC. The e-Synthesis app (version 1.9.8, a survey tool; Sleman, Yogyakarta, Indonesia) was purposefully developed by the e-HDSS team exclusively for this study [21]. The respondents’ responses were directly entered into the e-Synthesis application and then uploaded to the Sleman HDSS server. Next, the data management team downloaded the data from the server to conduct the data cleaning and analysis. All the research data was securely stored on both the server and a computer with limited access.

### Data analysis

Prior to the analysis, all variables were dichotomized for simplification into “yes/no” by using the lowest category (e.g., "no/never/hardly" = "no") versus all other categories ("yes") (see S1 Table for a list of variables and S2 Table for descriptive analysis of all study variables). BMI was categorized based on obesity status (< 27 kg/m^2^ = "no"; ≥ 27 kg/m^2^ = "yes"). We analyzed age, a continuous variable, using its mean value and standard deviation (SD). A two-tailed P-value of ≤ 0.05 was considered statistically significant.

Descriptive statistics were used to describe the characteristics of the study population based on anti-RBD-SARS-CoV-2 IgG status at baseline, across variables of demographics, smoking status, obesity status, COVID-19 vaccination status, comorbidities, history of symptoms, contact with COVID-19 patients, mobility, and preventive measures. Logistic regression analyses were then performed to examine associations between demographics, clinical status, social behaviors, and vaccination status and anti-RBD-SARS-CoV-2 IgG seropositivity adjusting for confounding variables (variables with p-values ≤ 0.05 in the descriptive analysis) using a stepwise method.

**Fig 2:**
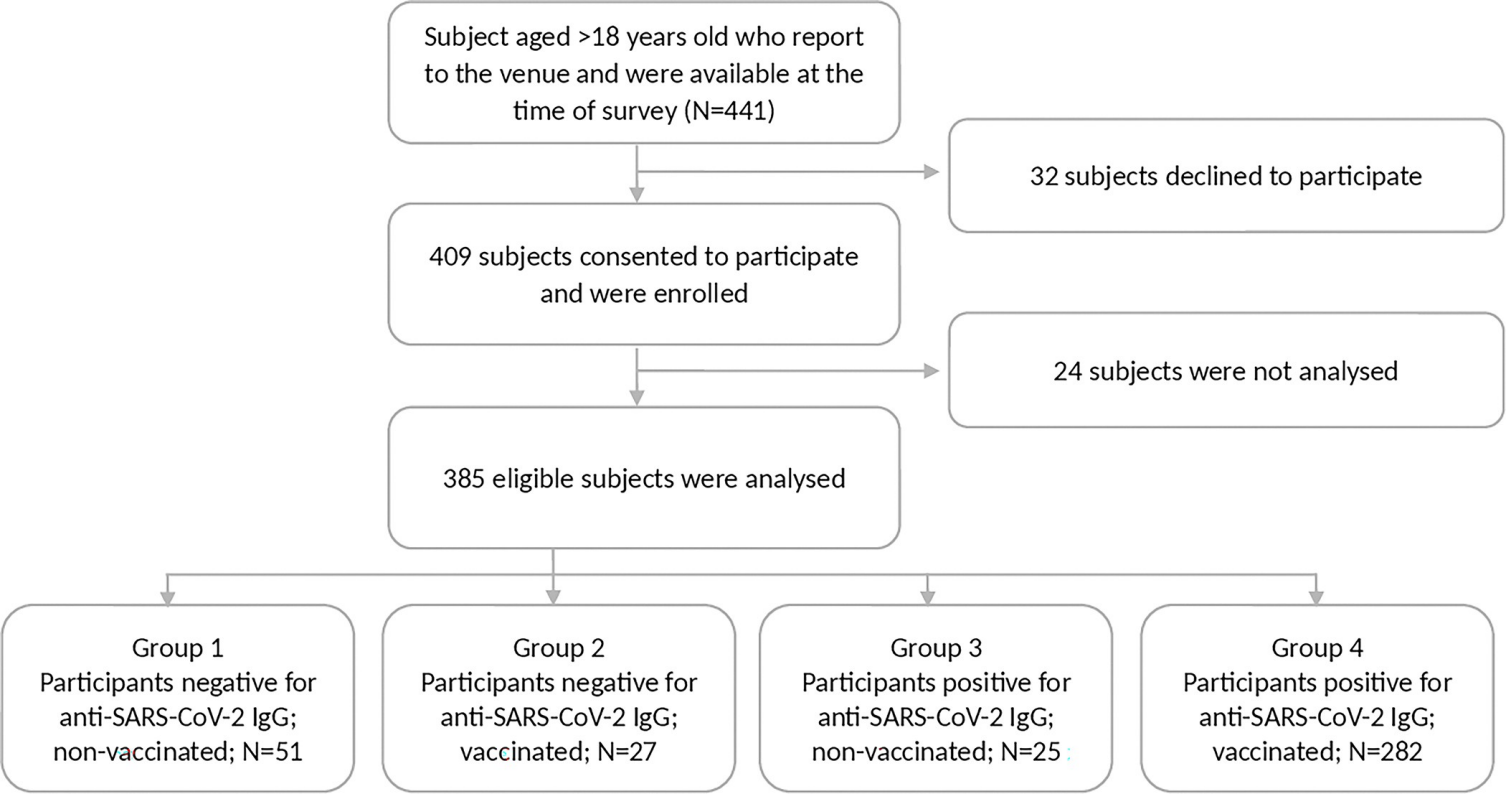
Inclusion of study participants and group analysis. Note: Of the 24 subjects not analyzed, 17 and 15 interviews and plasma data points were missing at monitoring 1 and 2, respectively.

Further descriptive analyses were conducted to compare subject characteristics across four groups defined by baseline vaccination status and IgG anti-RBD-SARS-CoV-2 seropositivity. Group 1 comprised of IgG seronegative-unvaccinated subjects; Group 2, seronegative-vaccinated subjects; Group 3, IgG seropositive-unvaccinated subjects; and Group 4, IgG seropositive-vaccinated subjects (see Fig 2).

The anti-RBD IgG antibody levels against SARS-CoV-2 at baseline, monitoring 1 (Mon-1; 4–5 weeks post-baseline), and monitoring 2 (Mon-2; 8–9 weeks post-baseline) were compared using the Wilcoxon signed-rank test, as well as to compare groups with and without PI vaccination during the monitoring period. These comparisons were conducted separately for the four groups defined by baseline vaccination status and anti-RBD-SARS-CoV-2 IgG seropositivity.

Statistical analyses were performed using Stata V.17.0 (Stata Corp LLC, College Station, TX, USA), and GraphPad Prism 9.0.0.121, for data visualization.

## Results

### Context of the study inclusion and vaccination program in Sleman District, Indonesia

This study was conducted from April 2021 to March 2022. It is important to note that at the time of study initiation (April 2021), the local government distributed the first doses of an inactivated whole-virus vaccine (Sinovac) to the general population. A national policy has been implemented for delivering the second dose to the expanded population as of August 2021. The vaccination rate for the first dose in Sleman District in December 2021 was 91.5% [18]. From July to October 2021, most COVID-19 cases in Sleman District and throughout Indonesia were primarily caused by the Delta strain [22–24]; furthermore, the Omicron strain dominated beginning in late November/early December 2021 [25]. All vaccinated subjects in this study received the vaccinations independently; thus, they could have been vaccinated at any time before and during study participation.

Additionally, the study was conducted during the period in which the Indonesian Government implemented Community Activities Restrictions Enforcement (CARE) to limit the transmission of SARS-CoV-2 and reduce COVID-19 morbidity. This restriction encompassed the closure of schools, the restriction of commercial service hours, the policy of working from home, and the tightening of personal prevention measures. The restriction was adjusted over time, by considering the transmission of SARS-CoV-2, and COVID-19 morbidity/ mortality.

Of the 441 healthy pre-selected participants who were available at the time of the survey and who came to the agreed-upon public health center, 32 (7.26%) had a positive GeNose C19^®^ test and were thus excluded. Twenty-four (24) subjects were lost to follow-up; hence, ultimately, 385 subjects were analyzed (Fig 2). Further analyses are described below.

The characteristics of the subjects (n = 385) based on their baseline anti-RBD-SARS-CoV-2 IgG level and seropositivity are shown in Table 1.

**Table 1:** Characteristics of the study population based on patients’ anti-RBD-SARS-CoV-2 IgG status at baseline.

Variables	Negative for anti-RBD-SARS-CoV-2 IgG (n = 78)	Positive for anti-RBD-SARS-CoV-2 IgG (n = 307)	Total (n = 385)	*p value*
	n (%)	n (%)	n (%)	
Age, mean (±SD)	51 (16.1)	50 (11.9)	50 (12.9)	0.72
Sex, female	41 (52.6)	210 (68.4)	251 (65.2)	**0.009**
Education level, >9 years	42 (53.8)	179 (58.3)	221 (57.4)	0.48
Job status, employed	49 (62.8)	158 (51.5)	207 (53.8)	0.07
Marital status, married	51 (65.4)	251 (81.8)	302 (78.4)	**0.002**
Smoking status, ever	23 (29.5)	52 (16.9)	75 (19.5)	**0.012**
Obesity status (≥27 kg/m^2^), yes^**a**^^**†**^	16 (21.1)	102 (33.2)	118 (30.8)	**0.040**
Vaccination status, yes^**†**^	27 (34.6)	282 (91.9)	309 (80.3)	**<0.0001**
Any comorbidity, yes^**†**^	16 (20.5)	95 (30.9)	111 (28.8)	0.07
History of any COVID-19 related symptoms, yes^**†**^	56 (71.8)	215 (70.0)	271 (70.4)	0.76
History of contact with people with COVID-19, yes^**†**^	7 (9.0)	34 (11.1)	41 (10.6)	0.59
History of going out, yes^**†**^	52 (66.7)	223 (72.6)	275 (71.4)	0.30
Mask and hand wash and social distancing, yes^**†**^	45 (57.7)	216 (70.4)	261 (67.8)	**0.033**

The data are n (%) unless noted otherwise.

^a^2 missing data points

^**†**^: see S1 Table. Study variables for explanation for dichotomization of categories

At baseline, 307 (79.7%) individuals tested positive for the IgG anti-RBD of SARS-CoV-2 antibody. We observed that sex, marital status, smoking habits, obesity, vaccination status and preventive measures were significantly different between the IgG anti-RBD SARS-CoV-2 seropositive and negative individuals (p≤ 0.05).

Further analysis to observe correlations of the variables with IgG seropositivity is shown in Table 2.

**Table 2:** Correlations among demographics, clinical status, possible transmission of infection, and vaccination status with anti-RBD-SARS-CoV-2 IgG seropositivity.

Variables	OR* [95% CI]	OR** [95% CI]	OR*** [95% CI]	OR**** [95% CI]	OR***** [95% CI]
Age, mean (±SD)	1.00 [0.98,1.02]	1.00 [0.98,1.02]	1.00 [0.98,1.02]	1.00 [0.98,1.02]	1.00 [0.98,1.02]
Sex, female	**1.95**^**a**^ **[1.18,3.24]**	**-**	-	-	-
Education level, >9 years	1.20 [0.73,1.98]	1.09 [0.65,1.82]	1.05 [0.62,1.76]	1.05 [0.62,1.77]	1.02 [0.60,1.73]
Employment status, employed	0.63 [0.38,1.05]	0.74 [0.43,1.29]	0.78 [0.44,1.36]	0.78 [0.45,1.37]	0.82 [0.46,1.44]
Marital status, married	**2.37**^**b**^ **[1.37,4.11]**	**-**	**-**	**-**	**-**
Smoking status, ever	**0.49**^**a**^ **[0.28,0.86]**	0.63 [0.29,1.36]	-	-	-
Obesity status (≥27 kg/m^2^), yes^**†**^	**1.87**^**a**^ **[1.02,3.40]**	1.51 [0.81,2.82]	1.48 [0.79,2.76]	1.5 [0.80,2.81]	-
Vaccination status, yes^**†**^	**21.31**^**c**^ **[11.46,39.62]**	**21.22**^**c**^ **[11.18,40.27]**	**21.03**^**c**^ **[11.08,39.93]**	**20.58**^**c**^ **[10.82,39.15]**	**19.87**^**c**^ **[10.39,38.00]**
Any comorbidity, yes^**†**^	1.74 [0.95,3.17]	**1.85**^**a**^ **[1.00,3.42]**	1.78 [0.96,3.30]	1.79 [0.96,3.33]	1.65 [0.88,3.10]
History of any COVID-19 related symptoms, yes^**†**^	0.92 [0.53,1.59]	0.87 [0.49,1.54]	0.89 [0.50,1.57]	0.90 [0.51,1.59]	0.91 [0.51,1.61]
History of contact with people with COVID-19, yes^**†**^	1.26 [0.54,2.97]	1.18 [0.49,2.83]	1.23 [0.51,2.97]	1.27 [0.52,3.08]	1.25 [0.51,3.03]
History of mobility, yes^**†**^	1.33 [0.78,2.26]	1.50 [0.86,2.60]	1.49 [0.86,2.59]	1.53 [0.88,2.67]	1.65 [0.94,2.89]
Preventive measures, yes^**†**^	1.74^a^ [1.04,2.90]	1.56 [0.92,2.65]	1.55 [0.91,2.63]	-	-

OR (odds ratio) was calculated by logistic regression for the unadjusted model

(*), adjusted for sex and marital status

(**), adjusted for sex, marital status and smoking status

(***), adjusted for sex, marital status, smoking status, and preventive measures

(****), and adjusted or sex, marital status, smoking status, obesity status and preventive measures

(*****). P <0.05 (a); P<0.01 (b); P<0.001 (c).

^**†**^: see S1 Table. Study variables for explanation for dichotomization of categories.

Overall, we identified that the best model to observe the correlation between variables and anti-RBD-SARS-CoV-2 IgG positivity at baseline was by including sex, marital status, smoking status, and preventive behaviours. By using this model, vaccination was the only variable that correlated with anti-RBD-SARS-CoV-2 IgG seropositivity [OR = 20.58; 95% CI 10.82, 39.15].

Based on the strong correlation between vaccination and anti-RBD-SARS-CoV-2 IgG seropositivity at baseline, we further analyzed all variables comparing anti-RBD-SARS-CoV-2 IgG seropositivity in vaccinated and unvaccinated group at baseline (Group 1 vs Group 3; and Group 2 vs Group 4). Table 3 shows the descriptive analysis.

**Table 3:** Overall characteristics by group of study participants by unvaccinated and vaccinated group status on baseline.

Variables	Group 1 (n = 51)	Group 2 (n = 27)	Group 3 (n = 25)	Group 4 (n = 282)	Total (n = 385)	P-value^b^	P-value^c^
	n (%)	n (%)	n (%)	n (%)	n (%)		
Age, mean (±SD)	48 (14.9)	57 (16.9)	49 (11.4)	50 (12.0)	50 (12.9)	0.61	**0.01**
Sex, female	30 (58.8)	11 (40.7)	16 (64.0)	194 (68.8)	251 (65.2)	0.66	**0.003**
Education level, >9 years	30 (58.8)	12 (44.4)	10 (40.0)	169 (59.9)	221 (57.4)	0.12	0.12
Employment status, employed	30 (58.8)	19 (70.4)	18 (72.0)	140 (49.6)	207 (53.8)	0.26	**0.04**
Marital status, married	33 (64.7)	18 (66.7)	18 (72.0)	233 (82.6)	302 (78.4)	0.53	**0.04**
Smoking status, ever	13 (25.5)	10 (37.0)	6 (24.0)	46 (16.3)	75 (19.5)	0.89	**0.015** ^***d***^
Obesity status (≥27 kg/m^2^), yes^a^^**†**^	9 (18.4)	7 (25.9)	10 (40.0)	92 (32.6)	118 (30.8)	**0.04**	0.48
Vaccination status, yes^**†**^	0 (0.0)	27 (100)	0 (0.0)	282 (100)	309 (80.3)	-	-
Any comorbidity, yes^**†**^	9 (17.6)	7 (25.9)	9 (36.0)	86 (30.5)	111 (28.8)	0.08	0.62
History of any COVID-19 related symptoms, yes^**†**^	38 (74.5)	18 (66.7)	20 (80.0)	195 (69.1)	171 (44.4)	0.60	0.79
History of contact with people with COVID-19, yes^**†**^	4 (7.8)	3 (11.1)	7 (28.0)	27 (9.6)	41 (10.6)	**0.034** ^***d***^	0.74^***d***^
History of mobility, yes^**†**^	33 (64.7)	19 (70.4)	18 (72.0)	205 (72.7)	275 (71.4)	0.53	0.80
Preventive measures, yes^**†**^	26 (51)	19 (70.4)	17 (68.0)	199 (70.6)	261 (67.8)	0.16	0.98

The data are n (%) unless noted otherwise.

^a^2 missing data points

^b^P*-*values for Group 1 and Group 3 calculated using chi-square tests

^c^P*-*values for Group 2 and Group 4 calculated using chi-square tests

^d^P*-*values calculated using Fisher’s exact test

^**†**^: see S1 Table. Study variables for explanation for dichotomization of categories.

Table 3 shows the differences in IgG seropositivity among those who were unvaccinated (Group 1 vs. Group 3) and vaccinated (Group 2 vs. Group 4) at inclusion. In the unvaccinated group, the proportion of individuals who were obese (P = 0.04) and had a history of contact with people with COVID-19 was greater among those who were IgG seropositive (P = 0.034). As for those who were vaccinated, the proportions of individuals who were younger, female, unemployed, married, and never smokers were greater among those with IgG seropositivity (P<0.05) than among those with IgG seronegativity.

**Fig 3:**
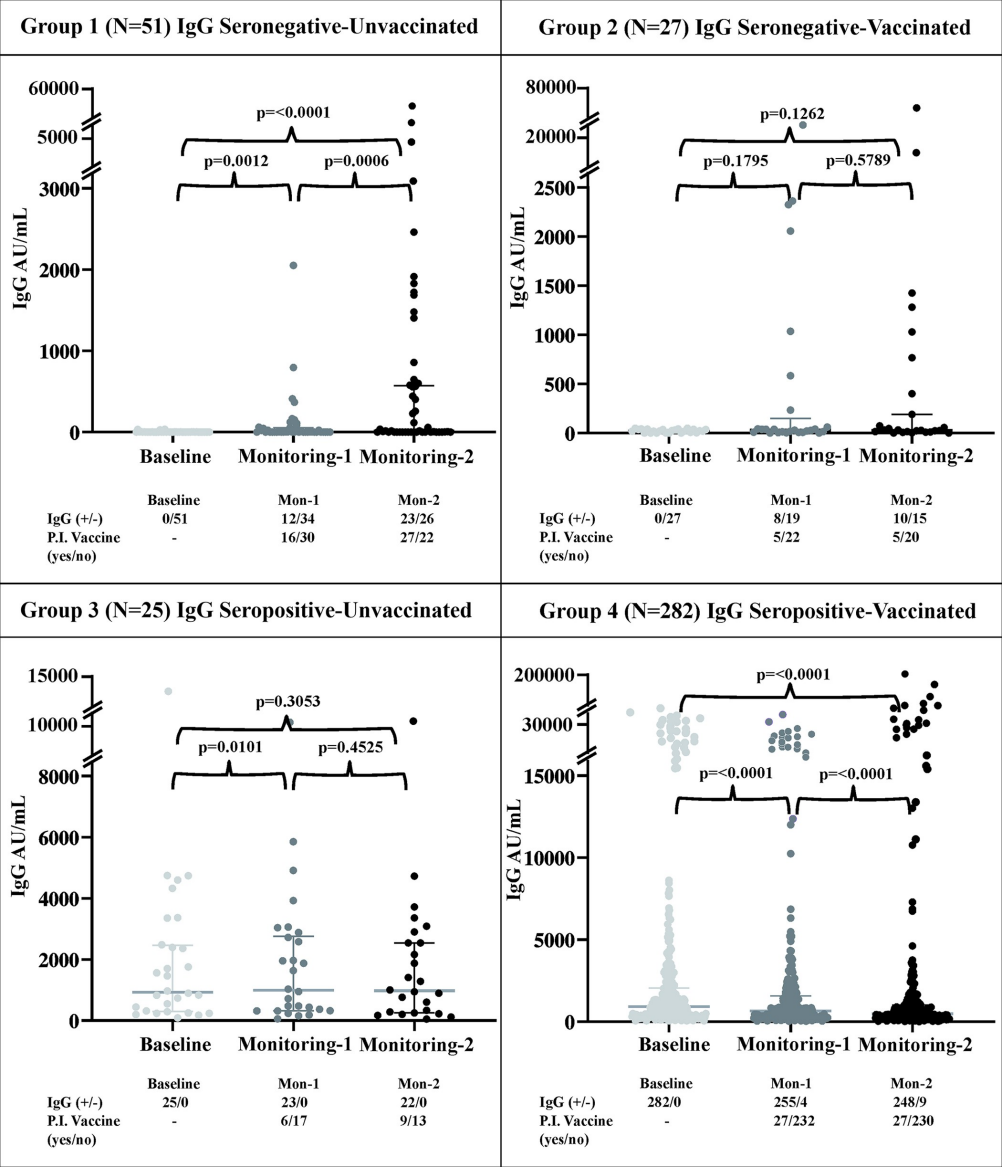
Comparison of anti-RBD IgG antibody levels against SARS-CoV-2 at baseline, monitoring 1 (Mon-1; 4–5 weeks post-baseline) and monitoring 2 (Mon-2; 8–9 weeks post-baseline) in the population in Sleman District, Indonesia. The numbers of subjects with IgG anti-RBD SARS-CoV-2 seropositivity (+/-) and postinclusion (PI) vaccination status (“yes/no”) at each data sampling point are depicted underneath each graph. Anti-RBD-SARS-CoV-2 IgG seropositivity = ≥ 50 AU/mL; the P-values for each monitoring session were calculated using the Wilcoxon matched-pair signed rank test. P≤ 0.05 was considered to indicate statistical significance.

Overall, we observed that seroprevalence in our cohort were 307/385 (79.74%), 268/325 (82.46%), and 303/353 (85.83%) on inclusion, monitoring 1 and 2, subsequently (see Fig 3 and S3 Table). Furthermore, the distributions of the anti-RBD-SARS-CoV-2 IgG titer in the four groups at baseline and at the subsequent monitoring sessions were analyzed (Fig 3). When comparing seroconversion at baseline and at monitoring 2, overall seroconversion from seronegativity to seropositivity was observed in Group 1 (23/51; 45.09%) and Group 2 (10/25; 40%), while seroconversion from seropositivity to seronegativity was observed in Group 4 (9/257; 3.50%). Additionally, all the subjects in Group 3 remained seropositive during this study. Group 1 exhibited a significant increase in the median IgG titer at monitoring 1 and 2 (P≤0.05). Group 3 showed a significant increase in IgG levels at monitoring session 1, in contrast to Group 4, in which the median IgG level decreased at monitoring 1 and 2 compared to baseline, respectively.

Regarding vaccination status, we observed that 1 or 2 vaccine doses were received in each group (Fig 3) during this study. By monitoring-2, among those who were not vaccinated at inclusion, in Group 1, sixteen (16) subjects had received one dose and 11 had received two doses of vaccination; in Group 3, three (3) subjects had received one dose and 7 subjects had received two doses. Among those who were vaccinated at inclusion, most had already received 2 doses (21/27 subjects; 77.8% and 251/282 subjects; 89% in Groups 2 and 4, respectively). By the end of monitoring, 5 and 27 subjects having received the second dose, in Group 2 and 4 respectively; leaving 1 and 4 subjects in Group 2 and 4, respectively with no additional vaccine (see S4 Table. Numbers of vaccination at inclusion, monitoring-1 and monitoring-2 among four different groups).

Furthermore, an analysis was performed to distinguish the IgG SARS-CoV-2 profile among those who received and did not receive postinclusion (PI) vaccination. PI vaccination was considered different among those who were unvaccinated (Groups 1 and 3) and vaccinated (Groups 2 and 4) at inclusion. Among those in Groups 1 and 3, 1–2 doses of the vaccine were administered during the monitoring period, while in Groups 2 and 4, some of the subjects received the second dose during monitoring period. Hence, by the end of this study, 10.65% (41/385), 4.95% (19/385), and 85.68% (324/385) of the participants had 0, 1, and 2 doses of vaccine, respectively (Fig 3).

**Fig 4:**
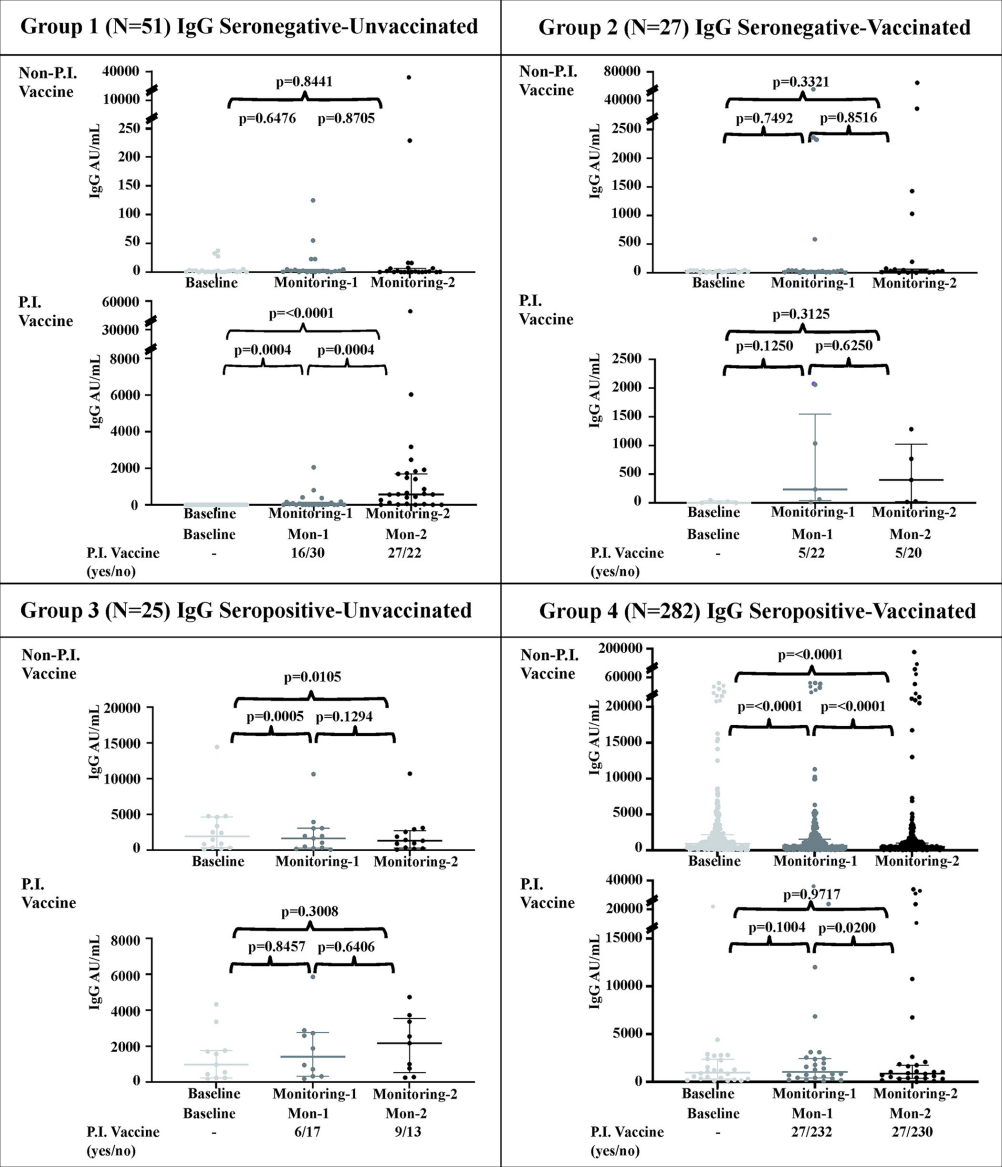
Comparison of groups without and with postinclusion (PI) vaccination during monitoring 1 (4–5 weeks PI) and monitoring 2 (8–9 weeks) in the four groups. Within Groups 2 and 4, PI vaccination included those who received second dose of the vaccine, while in Groups 1 and 3, PI vaccination consisted of 1–2 doses of the vaccine. The median of P-value was analyzed the Wilcoxon matched-pair signed rank test, and P≤0.05 was considered to indicate statistical significance.

Fig 4 shows that, in general, among those who were seronegative (Groups 1 and 2) and did not receive PI vaccination, they mostly remained seronegative (median values of 1.5 to 0.95 (P = 0.8441) and 25.7 to 29.55 (P = 0.3321), in group 1 and 2 respectively), while among those who were seropositive (Groups 3 and 4), a decrease in the median IgG titer was observed (median values of 1928 to 1284 (P = 0.0105) and 1003 to 523.7 (P<0.0001), in group 3 and 4 respectively). On the other hand, the group of subjects who received PI vaccination showed a trend toward an increase in the median IgG titer (median values of 0.3 to 579 (P<0.0001), 0.00–401.4 (P = 0.3125), and 971.3 to 2163 (P = 0.3008) in group 1,2, and 3 respectively), except slight decrease of IgG value for Group 4 (median value of 1067 to 1021; P = 0.9717).

## Discussion

Serosurveillance may be useful for determining the burden of COVID-19 in the general population. It has been reported that within two weeks after infection, IgM, IgG and IgA antibodies against SARS-CoV-2 are detected in the bloodstream, followed by the decay of IgM and IgA, while IgG is detected for weeks and months [27]. Vaccination against SARS-CoV-2 has been accepted as a key factor for ending the COVID-19 pandemic. In Indonesia, a national vaccination program against SARS-CoV-2 was launched on January 13, 2021. At the beginning of this study, 91.5% [18] of the population in Sleman District had received at least the first dose of the vaccine, as indicated in Fig 1. Notably, the inactivated COVID-19 vaccine (SINOVAC) was the only vaccine provided for the mass population in Indonesia at the time of this study; and it induced the production of anti-RBD antibodies [28–30]; therefore, anti-RBD IgG can be utilized to detect antibodies generated by natural infection or by vaccination within the scope of our study.

We also noted that from July to October 2021, most COVID-19 cases in Sleman District, as well as throughout Indonesia, were primarily caused by the Delta strain [23–25], which is associated with a higher transmission rate than the original strain and high clinical mortality. The high transmission and mortality rate of the Delta strain was the main reason for the long period of subject inclusion, at which point the Omicron peak occurred. Compared to the Delta strain, the Omicron strain is characterized by a greater transmission rate but much milder clinical symptoms and mortality. This may have led to the relaxation of health-related prevention measures at the community level; thus, potential changes in the perception of mobility or personal protection might have occurred.

Our study showed that by the end of February/March 2022, the seroprevalence of SARS-CoV-2 antibodies in the Sleman population was 79.7%, which was much greater than that reported in previous studies in Indonesia. Studies performed in Jakarta, Denpasar in Bali, Surabaya and Jombang in East Java, and Bantul District in Yogyakarta showed IgG prevalence rates of 28.52, 44.5, 11.4, and 31.1%, respectively [7–9, 31], which were much lower than the 79.7% in our study. All studies except that by Ahmad et al. [9] were conducted prior to the introduction of the vaccine. Additionally, only 3.05% of subjects recruited in the study by Ahmad et al. [9] had received their first dose of vaccine, whereas 86.5% of our participants received at least the first dose. This supports our finding that vaccination was the strongest driver of seroprevalence [OR = 20.58; 95% CI 10.82, 39.10]. Ours and other Indonesian studies agree that the seroprevalence was greater among adult as they have a higher potential to be exposed to SARS-CoV-2 either naturally or through vaccination. Factors that may contribute to the differences in seroprevalence and titers are differences in the methods and biomarkers used for seroprevalence status, as well as differences in the prevalence of the dominant SARS-CoV-2 strain during this study. Zaballa et al. [32] reported a serosurvey during the Omicron peak in Geneva, Switzerland, it showed a lower neutralizing capacity against Omicron than against Alpha strain (79.5 and 46.7%, respectively).

Severe COVID-19 is thought to trigger an earlier and more intense immune response in hospitalized patients [3, 5, 33]. The IgG titer is believed to decrease substantially more than 7 months after discharge from the hospital [34] as natural infection-induced seroconversion occurs. On the other hand, individuals with subclinical or asymptomatic responses may exhibit a low response or no seroconversion, as demonstrated in Group 1. This particular group may also consist of subjects who could have been unexposed and thus did not seroconvert. The main sources of the natural SARS-CoV-2 antigen were exposure to/contact with those who had COVID-19, as observed in Group 3 of our study. Seropositive individuals due to natural infection had higher antibody titers after vaccination compared to those who were vaccinated (see Fig 4, Group 3). We observed a decrease in the IgG titer in those who had been vaccinated 2 times (see Fig 4, Group 4), and 3.2% of individuals were seronegative at the end of this study. Antibody decay times differ between one vaccine and another, as do population characteristics. In our study, the longest possible duration since the second dose of the vaccine for participants in Group 4 was estimated to be 9 months (April 2021 to January 2022). Research in Aceh, Indonesia, showed that the total anti-SARS-CoV-2 RBD antibody titer decreased five months after the second dose of the Sinovac vaccine [35]. Furthermore, Sughayer et al. [36]. reported that the reduction of the IgG RBD neutralizing antibody level was reduced faster in patients who were vaccinated with an inactivated vaccine than in those who received the mRNA-based SARS-CoV-2 vaccine. We acknowledge that our monitoring period was shorter than that of the two references [35, 36] but agree that anti-SARS-CoV-2 antibody levels tended to decrease even during this short time. This calls for further booster doses.

Nonresponders, subjects who did not exhibit seroconversion, had low antibody titer, or experienced fast-decaying antibodies; showed no effective humoral immune response [37, 38] in our study (Group 2). This group was characterized by older age (57±16.9 vs. 50 ±12.0 years) compared to those with seropositive IgG (Group 4), despite receiving two doses of the vaccine. Studies have reported that nonresponder generally include infants, young children, elderly people, and immunocompromised patients [32, 39–41]. However, it is known that humoral immune response is not the only immune protection mechanism against viral infection [42, 43]. For nonresponders, different strategies should be developed to prevent morbidity when there is an endemic outbreak. For immunocompromised individuals, vaccination and measures to prevent viral transmission are essential to maintain antibody protection [44].

This study has various limitations. The number of subjects recruited was small, particularly when correlation analysis was done, showing the wide 95% convidence interval of COVID-19 vaccination data and IgG seropositivity [OR = 20.58; 95% CI 10.82, 39.15]. We asked about major signs and symptoms instead of COVID-19 history to avoid the negative stigma associated with COVID-19 status. Despite the short duration of the monitoring, the study’s recruitment window was broad, allowing for the dynamic nature of the SARS-CoV-2 variant, the dynamic of the transmission, changes in local governmental policies, and changes in the target population’s behavior, thereby making the study challenging.

To our knowledge, this is the first prospective longitudinal study to describe the role of hybrid immunity in shaping the dynamics of SARS-CoV-2 antibodies in a general population in Indonesia; given the seroprevalence profile, findings on seroconversion, and hybrid immunity, these conclusions are relevant to other findings. The nonresponder identified after COVID-19 vaccination should receive health recommendations, especially during endemic outbreaks.

## Conclusion

In this study, we found that COVID-19 vaccination was the main driver for seroconversion in Sleman. We found a small proportion of individuals who did not seroconvert following two doses of COVID-19 vaccination, particularly older adults, and also a small proportion whose antibodies have waned to undetectable after eight months. These suggest the need for a booster COVID-19 vaccination. Our data has implications for the Indonesian government regarding COVID-19 vaccination and highlights the need for continued surveillance of COVID-19 population immunity.

## Supporting information

S1 Table Study variables. (DOCX)

S2 Table Descriptive analysis of all study variables. (DOCX)

S3 Table Seroprevalence of study populations at the baseline, monitoring-1 and monitoring-2. (DOCX)

S4 Table Numbers of vaccination at inclusion, monitoring-1 and monitoring-2 among four different groups. (DOCX)

S1 File (DOCX)
