# Supplementary material for: Seroconversion and dynamics of IgG anti-SARS-CoV-2 antibodies during the pandemic: A two-month observation cohort study on the population of Sleman in Indonesia
Source: PLoS One. 2025 Jan 2;20(1):e0316360. doi: 10.1371/journal.pone.0316360 (PMC11695021; doi:10.1371/journal.pone.0316360)
Supplement: S1 Table — (DOCX) [file pone.0316360.s001.docx]

**Supporting information**

**S1 Table. Study variables**

| **No** | **Questions** | **Value** | **Value Label** | **Dichotomous variables** |
| --- | --- | --- | --- | --- |
| 1 | Respondent's name |  |  |  |
| 2 | Sex | 1 2 | Male Female |  |
| 3 | date of birth |  |  |  |
| 4 | Age (automatic generation by the app) |  |  | **Age:** mean |
| 5 | Marital status | 1 2 3 4 98 | 1. Not married 2. Married 3. Divorce 4. Widow/widower 98. Don’t Know | **Marital status:** 0: not married  1: any other |
| 6 | Highest/ current education | 1 2 3 4 5 6 7 98 | uneducated Basic school, 6 years Junior high, 3 years Senior high, 3 years Bachelor, 2-3 years University, 4-5 years Postgraduate Unknown | **Education level** ≤9 years (not yet at school - SMP) >9 years (SMA - S2/S3) |
| 7 | Main occupation | 1 2 3 4 5 6 7 8 9 1 11 12 13 14 15 16 17 18 19 2 21 22 | Opticians Osteopaths Pharmacists Pharmacy assistants Teacher assistants Nurses/physician’s assistants Care assistants and caregivers Sales and retail assistants Domestic assistants Association of health professionals Midwives Farm laborers Doctors Physiotherapists Teachers Special needs education teachers/SLB Elementary/Junior High School Teachers High School Teachers Kindergarten/PAUD teachers Cashiers  Pharmacy managers Managers in distribution, storage, and retail | **Job status** **Not employed/retired:** 81 82 85 91  **Employed:** 9 12 56 84 36 49 60 83 87 89 90 15 17 18 19 37 39 41 52 74 86 88 |
|  |  | 23 24 25 26 27 28 29 30 31 32 33 34 35 36 37 38 39 40 41 42 43 44 | Managers in the fields of animal husbandry, forestry, and fisheries Retail and wholesale managers Security managers Social service managers Childcare managers Storage and warehouse managers Farm manager Care and health practice managers Hospital and healthcare managers Transportation and distribution managers Food, beverage, and tobacco process operators Rail transport operators Paramedics  Food vendors Shop workers/employees Workers of welfare associations/social institutions Social workers Workers at train stations Protection service workers  Worker who packs/stacks items on store shelves Plantation workers Agricultural and fishery workers |  |
|  |  | 45 46 47 48 49 50 51 52 53 54 55 56 | Postal workers, mail sorters  Youth and Community Supporters  Window cleaners  Bakers, flour confectioners  Wholesaler/retail store owners and dealers Nannies  Bus drivers and helper  Heavy goods vehicle drivers  Train drivers  Van drivers Mortuary administrators and assistants/mortuary caretakers  Garbage or second-hand collectors |  |
|  |  | 57 58 59 60 61 62 63 64 65 66 67  68  69 70 71 72 73 74 75 76 77 78 79 80 | Fish seller, poultry cutter  Street sweepers  Nurses  Farmers  Social home office officers  Cleaners Police officers (sergeants and below)  Police officers (inspectors and above)  Prison service officer (under the main officer)  Probationary officers  Firefighters (firefighters and below)  Senior officers in firefighting, ambulances, prisons, and related services  Hospital Porters  Psychologists  Radiographers  Clergy  Medical secretaries  Drivers Ambulance staff Medical and dental technicians  Therapists  Speech and language therapists  Occupational therapists  Butchers |  |
|  |  | 81 82 83 84 85 86 87 88 89 90 91 95 | Housewives Students Self employed Laborer Pensioners Employees Merchants Civil servants Breeders Fishermen Not working Other, mention |  |
| 8 | Have you ever been diagnosed with Diabetes Mellitus by health workers (doctors, nurses, midwives)? | 1 2 | yes no | **Any Comorbidity**  No: no comorbid Yes: at least one comorbid |
| 9 | Have you ever been diagnosed with a stroke by health workers (doctors, nurses, midwives)? | 1 2 | yes no |  |
| 10 | Have you ever been diagnosed with hypertension by health workers (doctors, nurses, midwives)? | 1 2 | yes no |  |
| 11 | Have you ever been diagnosed with a heart disease by health workers (doctors, nurses, midwives)? | 1 2 | yes no |  |
| 12 | Have you ever been diagnosed with asthma/wheezing/COPD by health workers (doctors, nurses, midwives)? | 1 2 | yes no |  |
| 13 | Have you ever been diagnosed with tuberculosis, (the test is that your doctor has examined your sputum by taking a sample of mucus released by coughing and sending it to a laboratory for analysis or X-ray (X-ray) of your chest) | 1 2 | yes no |  |
| 14 | Have you ever been diagnosed with a kidney disease by health workers (doctors, nurses, midwives)? | 1 2 | yes no |  |
| 15 | Have you ever been diagnosed with chronic liver disease (cirrhosis) by health workers (doctors, nurses, midwives)? | 1 2 | yes no |  |
| 16 | Weight |  |  | **BMI: w**eight/(height)^2 **Non-obese: <27 Kg/M^2 Obese:** ≥27 Kg/M^2 |
| 17 | height |  |  |  |
| 18 | Have you smoked in the last month? | 1 2 3 4 5 | Yes, every day  Yes, sometimes No, but I used to smoke every day No, but I used to smoke not every day/sometimes Never at all | **Smoking status** Ever/current: 1 - 4  Never: 5 |
| 19 | In the past month, when compared to the previous month, how often have you gone shopping? | 1 2 3 4 | Become never Becoming less frequent It's the same Become more frequent | **History of mobility** 0: become never-less frequent  1: it's the same-become more frequent |
| 20 | In the past month, when compared to the previous month, how often have you gone for a leisurely walk (around the house or park) ? | 1 2 3 4 | Become never Becoming less frequent It's the same Become more frequent |  |
| 21 | In the past month, when compared to the previous month, how often have you met more than 5 people outside the home? | 1 2 3 4 | Become never Becoming less frequent It's the same Become more frequent |  |
| 22 | In the past month, when compared to the previous month, how often have you visited a family who is not in the same house as you? | 1 2 3 4 | Become never Becoming less frequent It's the same Become more frequent |  |
| 23 | In the past month, how often have you worn a mask when going outdoors/to public places? | 1 2 3 4 | Always Often Sometimes Never | **Protective measurements** No: never-sometimes Yes: often-always |
| 24 | In the past month, how often have you kept your distance from others when outside your home/public places? | 1 2 3 4 | Always Often Sometimes Never |  |
| 25 | In the past month, how often have you washed your hands with soap and water for at least 20 seconds? | 1 2 3 4 | Always Often Sometimes Never |  |
| 26 | In the past 30 days, have you been in close contact (e.g. close proximity/face-to-face within 1 meter, holding hands/shaking hands) with anyone who is sick with symptoms that can be associated with COVID-19 illness, such as cough, fever, or difficulty breathing? | 1 2 | yes no | **History of contact with people with COVID-19**  No: answering "no" to both questions  Yes: answering "yes" to at least one question |
| 27 | In the past 30 days, have you had close contact (e.g. close proximity/face-to-face within 1 meter, holding hands/shaking hands) with a confirmed COVID-19 person or a patient under surveillance? | 1 2 | yes no |  |
| 28 | In the past month, have you had flu-like symptoms such as fever? | 1 2 | yes no | **History of any COVID-19 related symptoms**  No: No symptoms whatsoever Yes: At least have one of the symptoms |
| 29 | In the past month, have you had flu-like symptoms such as shortness of breath? | 1 2 | yes no |  |
| 30 | In the past month, have you had flu-like symptoms such as nausea? | 1 2 | yes no |  |
| 31 | In the past month, have you had flu-like symptoms such as malaise/fatigue (lethargy, weakness, weakness)? | 1 2 | yes no |  |
| 32 | In the past month, have you had flu-like symptoms such as coughing? | 1 2 | yes no |  |
| 33 | In the past month, have you had flu-like symptoms such as seizures? | 1 2 | yes no |  |
| 34 | In the past month, have you had flu-like symptoms such as skin disorders such as rashes? | 1 2 | yes no |  |
| 35 | In the past month, have you had flu-like symptoms such as vomiting? | 1 2 | yes no |  |
| 36 | In the past month, have you had flu-like symptoms such as muscle aches? | 1 2 | yes no |  |
| 37 | In the past month, have you had flu-like symptoms like a cold? | 1 2 | yes no |  |
| 38 | In the past month, have you had flu-like symptoms such as diarrhea? | 1 2 | yes no |  |
| 39 | In the past month, have you had flu-like symptoms such as odor complaints (not being able to smell)? | 1 2 | yes no |  |
| 40 | In the past month, have you had flu-like symptoms such as tasting complaints (not being able to taste sweet, bitter, salty, sour)? | 1 2 | yes no |  |
| 41 | In the past month, have you had flu-like symptoms such as headaches? | 1 2 | yes no |  |
| 42 | In the past month, have you had flu-like symptoms such as abdominal pain? | 1 2 | yes no |  |
| 43 | Have you ever been vaccinated against COVID-19? | 1 2 | yes no |  |
| 44 | How many times? | 1 2 | once twice |  |
| Note : translation is done through the use of excel translator from Bahasa Indonesia to English | | | |  |
