# Supplementary material for: Seroconversion and dynamics of IgG anti-SARS-CoV-2 antibodies during the pandemic: A two-month observation cohort study on the population of Sleman in Indonesia
Source: PLoS One. 2025 Jan 2;20(1):e0316360. doi: 10.1371/journal.pone.0316360 (PMC11695021; doi:10.1371/journal.pone.0316360)
Supplement: S2 Table — (DOCX) [file pone.0316360.s002.docx]

**Supporting information**

**S2 Table. Descriptive analysis of all study variables**

| **Variables** | **Negative for IgG anti RBD SARS-CoV-2**  **(n=78)** | **Positive for IgG anti RBD SARS-CoV-2**  **(n=307)** | **Total**  **(n=385)** | ***p value*** |
| --- | --- | --- | --- | --- |
| **Sex** |  |  |  |  |
| Male | 37(47.4) | 97(31.6) | 134(34.8) | 0.009 |
| Female | 41(52.6) | 210(68.4) | 251(65.2) |  |
| **Education Level** |  |  |  |  |
| Uneducated | 3(3.8) | 4(1.3) | 7(1.8) | 0.531 |
| Basic school, 6 years | 14(17.9) | 68(22.1) | 82(21.3) |  |
| Junior high, 3 years | 19(24.4) | 56(18.2) | 75(19.5) |  |
| Senior high, 3 years | 34(43.6) | 153(49.8) | 187(48.6) |  |
| Bachelor, 2-3 years | 4(5.1) | 11(3.6) | 15(3.9) |  |
| University, 4-5 years | 4(5.1) | 14(4.6) | 18(4.7) |  |
| Postgraduate | 0(0) | 1(0.3) | 1(0.3) |  |
| **Job Status** |  |  |  |  |
| Not employed/retired | 29(37.2) | 149(48.5) | 178(46.2) | 0.072 |
| Employed | 49(62.8) | 158(51.5) | 207(53.8) |  |
| **Marital Status** |  |  |  |  |
| Not married | 11(14.1) | 13(4.2) | 24(6.2) | 0.003 |
| Married | 51(65.4) | 251(81.8) | 302(78.4) |  |
| Divorce | 1(1.3) | 5(1.6) | 6(1.6) |  |
| Widow/widower | 15(19.2) | 38(12.4) | 53(13.8) |  |
| **Smoking status** |  |  |  |  |
| Yes, every day | 9(11.5) | 33(10.7) | 42(10.9) | 0.002 |
| Yes, sometimes | 2(2.6) | 8(2.6) | 10(2.6) |  |
| No, but I used to smoke every day | 5(6.4) | 3(1) | 8(2.1) |  |
| No, but I used to smoke not every day/sometimes | 7(9) | 8(2.6) | 15(3.9) |  |
| Never at all | 55(70.5) | 255(83.1) | 310(80.5) |  |
| **Obesity Status** |  |  |  |  |
| Non-Obesity | 60(78.9) | 205(66.8) | 265(69.2) | 0.040 |
| Obesity | 16(21.1) | 102(33.2) | 118(30.8) |  |
| **Vaccination status** |  |  |  |  |
| No | 51(65.4) | 25(8.1) | 76(19.7) | 0.000 |
| Yes | 27(34.6) | 282(91.9) | 309(80.3) |  |
| **Comorbidity** | | | | |
| **Diabetes Mellitus** |  |  |  |  |
| No | 77(98.7) | 295(96.1) | 372(96.6) | 0.251 |
| Yes | 1(1.3) | 12(3.9) | 13(3.4) |  |
| **Stroke** |  |  |  |  |
| No | 77(98.7) | 304(99) | 381(99) | 0.813 |
| Yes | 1(1.3) | 3(1) | 4(1) |  |
| **Heart Disease** |  |  |  |  |
| No | 75(96.2) | 300(97.7) | 375(97.4) | 0.437 |
| Yes | 3(3.8) | 7(2.3) | 10(2.6) |  |
| **Asthma/wheezing/COPD** |  |  |  |  |
| No | 76(97.4) | 293(95.4) | 369(95.8) | 0.430 |
| Yes | 2(2.6) | 14(4.6) | 16(4.2) |  |
| **Tuberculosis** |  |  |  |  |
| No | 78(100) | 304(99) | 382(99.2) | 0.381 |
| Yes | 0(0) | 3(1) | 3(0.8) |  |
| **Kidney Disease** |  |  |  |  |
| No | 76(97.4) | 300(97.7) | 376(97.7) | 0.882 |
| Yes | 2(2.6) | 7(2.3) | 9(2.3) |  |
| **Chronic Liver Disease (cirrhosis)** |  |  |  |  |
| No | 78(100) | 305(99.3) | 383(99.5) | 0.475 |
| Yes | 0(0) | 2(0.7) | 2(0.5) |  |
| **Hypertension** |  |  |  |  |
| No | 69(88.5) | 243(79.2) | 312(81) | 0.061 |
| Yes | 9(11.5) | 64(20.8) | 73(19) |  |
| **History of any COVID-19 related symptoms** | | | | |
| **Fever** |  |  |  |  |
| No | 73(93.6) | 271(88.3) | 344(89.4) | 0.174 |
| Yes | 5(6.4) | 36(11.7) | 41(10.6) |  |
| **Shortness of Breath** |  |  |  |  |
| No | 73(93.6) | 288(93.8) | 361(93.8) | 0.942 |
| Yes | 5(6.4) | 19(6.2) | 24(6.2) |  |
| **Nausea** |  |  |  |  |
| No | 72(92.3) | 269(87.6) | 341(88.6) | 0.245 |
| Yes | 6(7.7) | 38(12.4) | 44(11.4) |  |
| **Malaise/fatigue (lethargy, weakness, weakness)** |  |  |  |  |
| No | 52(66.7) | 220(71.7) | 272(70.6) | 0.387 |
| Yes | 26(33.3) | 87(28.3) | 113(29.4) |  |
| **Coughing** |  |  |  |  |
| No | 62(79.5) | 258(84) | 320(83.1) | 0.338 |
| Yes | 16(20.5) | 49(16) | 65(16.9) |  |
| **Seizures** |  |  |  |  |
| No | 77(98.7) | 303(98.7) | 380(98.7) | 0.988 |
| Yes | 1(1.3) | 4(1.3) | 5(1.3) |  |
| **Skin disorders such as rashes** |  |  |  |  |
| No | 74(94.9) | 285(92.8) | 359(93.2) | 0.522 |
| Yes | 4(5.1) | 22(7.2) | 26(6.8) |  |
| **Vomiting** |  |  |  |  |
| No | 75(96.2) | 294(95.8) | 369(95.8) | 0.878 |
| Yes | 3(3.8) | 13(4.2) | 16(4.2) |  |
| **Muscle aches** |  |  |  |  |
| No | 58(74.4) | 195(63.5) | 253(65.7) | 0.072 |
| Yes | 20(25.6) | 112(36.5) | 132(34.3) |  |
| **Cold** |  |  |  |  |
| No | 66(84.6) | 260(84.7) | 326(84.7) | 0.987 |
| Yes | 12(15.4) | 47(15.3) | 59(15.3) |  |
| **Diarrhea** |  |  |  |  |
| No | 74(94.9) | 290(94.5) | 364(94.5) | 0.887 |
| Yes | 4(5.1) | 17(5.5) | 21(5.5) |  |
| **Odor complaints (not being able to smell)** |  |  |  |  |
| No | 78(100) | 303(98.7) | 381(99) | 0.311 |
| Yes | 0(0) | 4(1.3) | 4(1) |  |
| **Tasting complaints (not being able to taste sweet, bitter, salty, sour)** |  |  |  |  |
| No | 78(100) | 303(98.7) | 381(99) | 0.311 |
| Yes | 0(0) | 4(1.3) | 4(1) |  |
| **Headaches** |  |  |  |  |
| No | 62(79.5) | 207(67.4) | 269(69.9) | 0.038 |
| Yes | 16(20.5) | 100(32.6) | 116(30.1) |  |
| **Abdominal pain** |  |  |  |  |
| No | 70(89.7) | 272(88.6) | 342(88.8) | 0.774 |
| Yes | 8(10.3) | 35(11.4) | 43(11.2) |  |
| **History of contact with people with COVID-19** | | | | |
| **Make a contact with probable COVID-19** |  |  |  |  |
| Yes | 5(6.4) | 25(8.1) | 30(7.8) | 0.610 |
| No | 73(93.6) | 282(91.9) | 355(92.2) |  |
| **Make a contact with confirmed COVID-19 cases** |  |  |  |  |
| Yes | 4(5.1) | 12(3.9) | 16(4.2) | 0.630 |
| No | 74(94.9) | 295(96.1) | 369(95.8) |  |
| **History of going out** | | | | |
| **Shopping** |  |  |  |  |
| Become never | 11(15.1) | 45(15.3) | 56(15.2) | 0.449 |
| Becoming less frequent | 38(52.1) | 128(43.4) | 166(45.1) |  |
| It's the same | 17(23.3) | 96(32.5) | 113(30.7) |  |
| Become more frequent | 7(9.6) | 26(8.8) | 33(9) |  |
| **Leisurely walk (around the house or park)** |  |  |  |  |
| Become never | 16(22.2) | 90(30.5) | 106(28.9) | 0.041 |
| Becoming less frequent | 22(30.6) | 88(29.8) | 110(30) |  |
| It's the same | 27(37.5) | 67(22.7) | 94(25.6) |  |
| Become more frequent | 7(9.7) | 50(16.9) | 57(15.5) |  |
| **Met more than 5 people outside the home** |  |  |  |  |
| Become never | 10(13.7) | 43(14.6) | 53(14.4) | 0.509 |
| Becoming less frequent | 39(53.4) | 132(44.7) | 171(46.5) |  |
| It's the same | 14(19.2) | 79(26.8) | 93(25.3) |  |
| Become more frequent | 10(13.7) | 41(13.9) | 51(13.9) |  |
| **Visited a family that is not in the same house** |  |  |  |  |
| Become never | 11(15.1) | 59(20.1) | 70(19.1) | 0.645 |
| Becoming less frequent | 45(61.6) | 176(59.9) | 221(60.2) |  |
| It's the same | 9(12.3) | 37(12.6) | 46(12.5) |  |
| Become more frequent | 8(11) | 22(7.5) | 30(8.2) |  |
| **Health behavior** | | | | |
| **Wearing a mask** |  |  |  |  |
| Always | 51(65.4) | 223(72.6) | 274(71.2) | 0.234 |
| Often | 18(23.1) | 63(20.5) | 81(21) |  |
| Sometimes | 9(11.5) | 18(5.9) | 27(7) |  |
| Never | 0(0) | 3(1) | 3(0.8) |  |
| **Keep distance from others** |  |  |  |  |
| Always | 38(48.7) | 177(57.7) | 215(55.8) | 0.538 |
| Often | 18(23.1) | 62(20.2) | 80(20.8) |  |
| Sometimes | 18(23.1) | 57(18.6) | 75(19.5) |  |
| Never | 4(5.1) | 11(3.6) | 15(3.9) |  |
| **Washing hands** |  |  |  |  |
| Always | 37(47.4) | 169(55) | 206(53.5) | 0.221 |
| Often | 25(32.1) | 93(30.3) | 118(30.6) |  |
| Sometimes | 13(16.7) | 42(13.7) | 55(14.3) |  |
| Never | 3(3.8) | 3(1) | 6(1.6) |  |
