# Supplementary material for: Seroconversion and dynamics of IgG anti-SARS-CoV-2 antibodies during the pandemic: A two-month observation cohort study on the population of Sleman in Indonesia
Source: PLoS One. 2025 Jan 2;20(1):e0316360. doi: 10.1371/journal.pone.0316360 (PMC11695021; doi:10.1371/journal.pone.0316360)
Supplement: S3 Table — (DOCX) [file pone.0316360.s003.docx]

**Supporting information**

**S3 Table. Seroprevalence of study populations at the baseline, monitoring-1 and monitoring-2**

|  |  | **Baseline** | |  | **Monitoring-1** | |  | **Monitoring-2** | |
| --- | --- | --- | --- | --- | --- | --- | --- | --- | --- |
| **Group** |  | N (sero+/-) | seroprevalence (%) |  | N (sero+/-) | seroprevalence (%) |  | N (sero+/-) | seroprevalence (%) |
| 1 |  | 0/51 | 0.00 |  | 12/34 | 26.09 |  | 23/26 | 46.94 |
| 2 |  | 0/27 | 0.00 |  | 8/19 | 29.63 |  | 10/15 | 40.00 |
| 3 |  | 25/0 | 100.00 |  | 23/0 | 100.00 |  | 22/0 | 100.00 |
| 4 |  | 282/0 | 100.00 |  | 225/4 | 98.25 |  | 248/9 | 96.88 |
| subtotal |  | 307/385 | 79.74 |  | 268/325 | 82.46 |  | 303/353 | 85.83 |
