# Supplementary material for: Seroconversion and dynamics of IgG anti-SARS-CoV-2 antibodies during the pandemic: A two-month observation cohort study on the population of Sleman in Indonesia
Source: PLoS One. 2025 Jan 2;20(1):e0316360. doi: 10.1371/journal.pone.0316360 (PMC11695021; doi:10.1371/journal.pone.0316360)
Supplement: S4 Table — (DOCX) [file pone.0316360.s004.docx]

**Supporting information**

**S4 Table. Numbers of vaccination at inclusion, monitoring-1 and monitoring-2 among four different groups**

|  |  |  |  |  |  |  |  |  |  |  |  |  |  |
| --- | --- | --- | --- | --- | --- | --- | --- | --- | --- | --- | --- | --- | --- |
|  |  | **Baseline (N=51)** |  |  |  | **Monitoring-1 (N=46)** |  |  |  |  | **Monitoring-2 (N=49)** |  |  |
| **Group** | **No. vaccination** | | |  | **No. vaccination** | | | **N.A** |  | **No. vaccination** | | | **N.A** |
| **1** | **0** | **1** | **2** |  | **0** | **1** | **2** |  |  | **0** | **1** | **2** |  |
|  | 51 | 0 | 0 |  | 30 | 12 | 4 | 5 |  | 22 | 16 | 11 | 2 |
|  |  | **Baseline (N=27)** |  |  |  | **Monitoring-1 (N=27)** |  |  |  |  | **Monitoring-2 (N=25)** |  |  |
|  | **No. vaccination** | | |  | **No. vaccination** | | |  |  | **No. vaccination** | | |  |
| **2** | **0** | **1** | **2** |  | **0** | **1** | **2** |  |  | **0** | **1** | **2** |  |
|  | 0 | 6 | 21 |  | 0 | 1 | 26 | 1 |  | 0 | 1 | 24 | 2 |
|  |  |  |  |  |  |  |  |  |  |  |  |  |  |
|  |  | **Baseline (N=25)** |  |  |  | **Monitoring-1 (N=23)** |  |  |  |  | **Monitoring-2 (N=22)** |  |  |
|  | **No. vaccination** | | |  | **No. vaccination** | | |  |  | **No. vaccination** | | |  |
| **3** | **0** | **1** | **2** |  | **0** | **1** | **2** |  |  | **0** | **1** | **2** |  |
|  | 25 | 0 | 0 |  | 17 | 6 | 0 | 2 |  | 13 | 3 | 6 | 3 |
|  |  |  |  |  |  |  |  |  |  |  |  |  |  |
|  |  | **Baseline (N=282)** |  |  |  | **Monitoring-1 (N=259)** |  |  |  |  | **Monitoring-2 (N=257)** |  |  |
|  | **No. vaccination** | | |  | **No. vaccination** | | |  |  | **No. vaccination** | | |  |
| **4** | **0** | **1** | **2** |  | **0** | **1** | **2** |  |  | **0** | **1** | **2** |  |
|  | 0 | 31 | 251 |  | 0 | 4 | 255 | 23 |  | 0 | 4 | 253 | 25 |
|  |  |  |  |  |  |  |  |  |  |  |  |  |  |

N.A: data not available / misssing
