# Supplementary material for: Seroconversion and dynamics of IgG anti-SARS-CoV-2 antibodies during the pandemic: A two-month observation cohort study on the population of Sleman in Indonesia
Source: PLoS One. 2025 Jan 2;20(1):e0316360. doi: 10.1371/journal.pone.0316360 (PMC11695021; doi:10.1371/journal.pone.0316360)
Supplement: S1 File — (DOCX) [file pone.0316360.s005.docx]

1. Before we proceed with your manuscript, please also provide non-author contact information (phone/email/hyperlink) for a data access committee, ethics committee, or other institutional body (e.g., Principal Investigator of the study and the Medical and Health Research Ethics Committee (MHREC), Faculty of Medicine, Public Health, and Nursing, Universitas Gadjah Mada) to which data requests may be sent to obtain the minimal data for your submission.

The minimal data access of this study can be requested by contact PI:

Eggi Arguni, MD, PhD.

Professor Pediatric Infectious Disease

email: eggiarguni@ugm.ac.id

telephone: +62 81578531122

Office address:

Department of Child Health, Faculty of Medicine, Public Health, and Nursing

Universitas Gadjah Mada

Jl. Farmako, Sekip Utara, Sleman

Yogyakarta 55262

INDONESIA

Or by contact Medical and Health Research Ethics Committee (MHREC), Faculty of Medicine, Public Health, and Nursing, Universitas Gadjah Mada.

Telp.+62-274-588688, ext. 17225

+62-8112666869

email: mhrec_fmugm@ugm.ac.id

To ensure persistent and long-term data storage and availability for the retrospective study using secondary data from hospital medical records, we are taking the following steps:

1. The data has been anonymized to protect patient privacy, ensuring that no personally identifiable information is included.

2. The anonymized data will be stored in a secure institutional data repository that complies with regulatory standards for data protection and privacy

3. Access to the data will be restricted to authorized personnel only, with necessary permissions and ethical approvals in place, to ensure that data is only used for legitimate research purposes.

4. Regular backups of the data will be performed to prevent data loss. These backups will be stored in different physical locations to add an extra layer of security.

5. By employing version control systems, we can ensure that the integrity of the data is maintained and any changes or updates are thoroughly documented.

6. We will collaborate with the IT department of the institution to ensure that the data storage and management practices align with the highest standards of data preservation and security.
